# 892 Tending to Smaller Burns: Evaluating Our 15% TBSA Burn Resuscitation Threshold

**DOI:** 10.1093/jbcr/iraf019.423

**Published:** 2025-04-01

**Authors:** Ashleigh Bull, Mala Sharma, Alexander Kurjatko, Colette Galet, Lucy Wibbenmeyer

**Affiliations:** University of Iowa Health Care Burn Center; University of Iowa Health Care Burn Center; University of Iowa Health Care Burn Center; University of Iowa Health Care Burn Center; University of Iowa Health Care Burn Center

## Abstract

**Introduction:**

The American Burn Life Support (ABLS) course recommends fluid resuscitation of patients with total burned surface area (TBSA) ≥20% to prevent burn shock. Very little attention is given to burns < 20% TBSA. Our center resuscitates patients with burns greater than 15% TBSA. Herein, we aimed to characterize those patients who received resuscitation with TBSA between 15-19%.

**Methods:**

Patients with burns 15 to 19% admitted from 1/1/2019 to 3/31/2023 were included if they received fluid resuscitation via Brooke, Parkland (PF), or other formula. Demographics, hospital course, and fluids received were reviewed. Fluid resuscitation was categorized as “below resuscitation range” (PF < 3mL/kg/%TBSA), “within resuscitation range” (PF = 3-5mL/kg/%TBSA or “above resuscitation range” (PF > 5mL/kg/%TBSA). Similarly, urine output (UOP) was expressed as “below goal UOP range” (< 30 mL/h), “within goal UOP range” (31-50 mL/h) or “above goal UOP range” (>50 mL/h). Fluid volumes were compared were considered “within maintenance range” for maintenance fluids if the volume fell within 10% of maintenance by ideal body weight. Categorical variables were compared using Chi-square and Fisher exact tests and continuous variables using the Mann-Whitney U test. P < 0.05 was considered significant.

**Results:**

Thirty-three patients with burns 15 to 19% received fluid resuscitation via Brooke (9.1%), PF (63.6%), or other formula (27.3%), median age was 57 years. Most were male (81.8%) with a TBSA of 17%. Only 15.2% had inhalation injuries and over two-thirds had comorbidities (69.7%). Four died, two from immediate complications, one from a cardiopulmonary complication, and one from a septic complication. Almost 20% of patients required vasopressors during resuscitation. Fifteen patients were within resuscitation range, 15 were below resuscitation range, and 3 were above resuscitation range. There was no significant difference between the PF groups with respect to demographics, burn injury variables, or complications. Those who were below resuscitation range had a shorter length of stay (LOS, 10 vs. 17 days, (p=0.033). The majority of those within and above resuscitation range had high UOP. Notably, the average creatinine 24 hours post-admission was 0.9 and average lactate was 2. There was no difference in ventilator days or mortality observed between the groups. Half of the study patients were above maintenance range.

**Conclusions:**

While not designed to show the efficacy of resuscitating burns < 20%, this retrospective study shows that patients with smaller burns do not seem to be harmed and may benefit from resuscitation as 50% required more than maintenance. Resuscitation of smaller burns requires more study.

**Applicability of Research to Practice:**

Patients with burns < 20% may benefit from resuscitation that is carefully monitored.

**Funding for the Study:**

N/A

